# Impact of Railway Delay Announcements on Suicide-Related Associations, Emotions, and Announcement Appreciation: A Replication Study

**DOI:** 10.1027/0227-5910/a001061

**Published:** 2026-06-12

**Authors:** Felix Bolinski, Stephanie Leone, Lonneke van Leeuwen, Jeroen Bommelé, Bart Hoogcarspel, Anne C. Miers

**Affiliations:** ^1^ Department of Mental Health & Prevention, Trimbos Institute – Netherlands Institute of Mental Health and Addiction, Utrecht, The Netherlands; ^2^ Global Public Health & Bioethics, Julius Center for Health Sciences and Primary Care, University Medical Centre Utrecht, Utrecht, The Netherlands; ^3^ Expertise Center for Tobacco Control (NET), Trimbos Institute – Netherlands Institute of Mental Health and Addiction, Utrecht, The Netherlands; ^4^ Cluster Safety, ProRail, Utrecht, The Netherlands

**Keywords:** suicide prevention, explicit associations, responsible communication, suicides on railways, announcement emotional impact

## Abstract

**Abstract:** *Background:* Previous research has showed that exposure to a postsuicide railway announcement stating that a delay was due to a collision with a person yielded suicide associations and negative emotions. In 2021, the Dutch Railways implemented a new announcement that simply stated a collision had occurred, but its impact has not yet been tested. *Aims:* This follow-up study investigates the impact of this new announcement on suicide-related explicit associations, negative and positive emotions, and announcement appreciation. We compare it to the previously used announcement collision with a person, as well as to announcements stating that a delay was due to the presence of emergency services and to a collision with an animal (control announcement). *Method:* A randomized controlled online experiment was conducted in which participants (*N* = 346) were exposed to one of eight conditions varying across two factors: announcement and personal relevance of the delay. Participants were recruited from the Dutch railway customer panel. *Results:* The new announcement stating that a delay was due to a collision was less likely to yield associations with suicide than the collision with a person announcement, but more likely to elicit suicide associations compared to the announcement indicating presence of emergency services. However, the latter was the least appreciated delay announcement. Regarding positive and negative emotions, the new collision announcement did not significantly differ from the other announcements. Personally relevant delays yielded greater suicide associations and stronger negative emotions than nonrelevant delays but more willingness to help other passengers. *Limitations:* The extent to which the scenarios used in the online experiment reflect real-life situations may be limited. *Conclusion:* Announcement of collision or emergency services is more appropriate than stating collision with a person in a delay announcement.

Suicide is a major public health concern, which beyond the direct loss of life can lead to copycat behavior (Walling, 2021). In 2023, more than 1,800 persons died by suicide in the Netherlands (Balt et al., 2024), of which roughly 10% were railway suicides (Statistics Netherlands [Centraal Bureau voor de Statistiek], 2025). Railway operators are therefore an important stakeholder when implementing and testing suicide prevention measures.

The main Dutch railway operator (Dutch Railways; [Nederlandse Spoorwegen, NS]), together with ProRail, the respective governmental infrastructure organization, have implemented a comprehensive package of measures to reduce the incidence of suicides. Among these are the restriction of access to railways by means of physical barriers, as well as experimental approaches, including blue lights that are installed at various high-risk locations throughout the country (Matsubayashi et al., 2013). First reports indicate that this comprehensive package might have led to a decrease of 30% in railway suicides (Van Houwelingen et al., 2021).

Despite the effects of such measures, many argue that suicide cannot be completely prevented (Pridmore & Pridmore, 2019), thereby emphasizing the need for an appropriate response to, and aftercare for, those affected by suicide (Andriessen et al., 2019). This approach is termed ‘postvention’ (Andriessen et al., 2019), which can support the further prevention of suicide for example by reducing copycat behavior (Jordan, 2017). Responsible reporting after a suicide (attempt) is one of the cornerstones of effective postvention strategies (Colman, 2018). These strategies typically caution against directly mentioning the method or location of suicide when communicating about an incident.

In this context, the delay caused by railway suicides requires train operators to provide a well-considered announcement for passengers waiting for their train, while reducing the association with suicide in other, potentially vulnerable passengers. In 2020, Van Leeuwen et al. published a study on the impact of three postrailway suicide delay announcements on suicide-related associations. The announcements stated that the delay was caused by a *collision with a person*, the presence of *emergency services* on the tracks, or a *collision with an animal*. The study found that participants exposed to the *collision with a person* announcement were about nine times more likely to assume suicide as the cause of delay relative to the *emergency services* announcement (van Leeuwen et al. 2020). A UK-based study also found that a message stating there had been a collision with a person increased associations with suicide compared to a message about emergency services (Majava & McNaughton Nicholls, 2015). The study also suggested that this finding may encourage railways to communicate delays due to suicide in ways that do not imply suicide by train. Consequently, ProRail and Dutch Railways implemented a new postrailway suicide delay announcement in their communication with passengers in all stations (2021). This announcement states that the delay is due to a *collision* and deliberately avoids specifying the cause of the collision, including whether it involved suicide.

The aim of the current study was to conduct a follow-up investigation to the van Leeuwen et al. (2020) study, whereby the impact of the new delay announcement on suicide-related explicit associations, emotions, and announcement appreciation was explored. We compared the new delay announcement to those previously tested. We also investigated how different announcements affect passengers’ emotional responses and the potential for aggressive behavior toward staff. To this end, we considered both negative emotions (frustration, anger) and positive emotions (empathy, willingness to help others). Based on previous findings, we hypothesized that:

Hypothesis 1 (H1):
the *collision with a person* announcement is more strongly associated with the concept of suicide than the announcements *collision*, *emergency services*, or *collision with an animal* (control announcement).

Hypothesis 2 (H2):
the emotional impact of the *collision with a person* announcement is stronger than that of *collision*, *emergency services*, or *collision with an animal*.

Hypothesis 3 (H3):
the personal relevance of the delay (i.e., does the delay disrupt the imagined travel plans) increases the impact of the delay announcement, such that those who are affected by the delay form stronger associations with the concept of suicide and have stronger emotional responses.

Hypothesis 4 (H4):
appreciation is higher for the announcements that include a specification of the reason for the delay, namely *collision with a person* and *collision with an animal* compared to announcements that do not specify a reason, namely *collision* and *emergency services*.

## Method

### Recruitment and Design

The design generally followed the original study (van Leeuwen et al., 2020). Approximately 1,000 participants were sampled from the Dutch Railways customer panel (∼70,000 members). Using a market research agency (https://mwm2.com/), an email was sent to the sample with an explanation of the study purpose (i.e., to improve communication on railway delays) and a link to an online survey. To avoid any prior activation of the concept of suicide, any suicide-related words or reference to suicide researchers were purposely provided only after completing the survey. Participants were given the option to consent to the use of their data in the study or to have it removed following disclosure at the end of the survey. They also received information on support services such as the Dutch suicide prevention helpline *113 Zelfmoordpreventie* and other mental health support sources. We did not include the implicit associations used previously because of the limited value of the results. Data shared with the Trimbos Institute (i.e., the research team) was completely anonymous. The Trimbos Institute’s Ethical Committee gave ethical approval for the study on April 23, 2025 (Reference 202506).

### Randomization and Procedure

Participants were randomized into one of eight conditions that varied by two factors: delay announcement and personal relevance of the delay. At the start of the survey, each participant was presented with a written scenario describing a train delay with an accompanying delay announcement (Table 1). In the current study, there were different delay announcements: *collision with a person*, *collision*, deployment of *emergency services*. *Collision with an animal* served as the control announcement. Each scenario could be either personally relevant (i.e., the delay hindered the participant’s imagined trip) or not personally relevant (i.e., the delay did not hinder the imagined trip). After reading the scenario and before proceeding to the survey items, participants were asked to indicate the extent to which they could imagine themselves in the presented scenario (0 = *not at all*, 10 = *very much so*). Then, they completed a manipulation check by indicating whether their train was delayed or not in the presented scenario.

**Table 1:** Overview of delay announcements

Announcement type	Announcement text
Collision with a person	Due to a collision with a person, the intercity train to Amsterdam will not be running from platform 7.
Collision	Due to a collision, the intercity train to Amsterdam will not be running from platform 7.
Emergency services	Due to the deployment of emergency services on the railway, the intercity train to Amsterdam will not depart from platform 7.
Collision with an animal	Due to a collision with an animal, the intercity train to Amsterdam will not be running from platform 7.
*Note*. All four delay announcements can vary on personal relevance. In the relevant condition, the participant is on the way to an important meeting in Amsterdam and is directly affected by the delay. In the nonrelevant condition, the participant is traveling to a different destination and is not affected by the delay.	

### Measures

#### Associations With Suicide

Participants were asked what the most likely cause of the delay described in the scenario was and given the following response options: (1) collision with an animal; (2) accidental collision with a person; (3) emergency services handling an accident; (4) terrorist attack; or (5) collision with a person who was on the tracks on purpose. A dichotomous variable was created to reflect associations with suicide, coded as 1 (*presence of association*) if participants chose the option *collision with a person who was on the tracks on purpose*, or 0 (*absence of association*) if any other option was chosen.

#### Emotional Impact

Emotional impact was assessed with eight items. Two items formed the factor *anger about the delay* (“I felt frustrated by the delay and I felt anger toward ProRail/Dutch Railways”; Cronbach’s *α* = .63). A factor *sadness about the incident* included two items (“I felt sad for the victim” and “The incident affected me”; Cronbach’s *α* = .80). The remaining four items were: “I felt anger toward the victim”; “I could accept the delay”; “I felt empathy for the railway staff”; “I was willing to support my fellow passengers.” Each item was rated on a scale of 0–10 (0 = *not at all*, 10 = *very much so*). Averaged composite scores were calculated for both factors.

#### Announcement Appreciation

The factor *announcement appreciation* (Cronbach’s *α* = .80) was created based on an averaged composite score of four items assessing whether the announcement was clear, honest, trustworthy, and informative. Each item was scored on scale of 0–10 (e.g., 0 = *unclear* to 10 = *clear*; or 0 = *dishonest* to 10 = *honest*).

#### Demographics

Age (in years) and gender (male, female, other, do not wish to disclose) were assessed.

### Statistical Analysis

Due to a zero cell count on one of the response options a loglinear analysis, as was done in the original study, could not be used. Instead, the differences between the conditions in suicidal associations (present: yes/no) were analyzed using a Firth penalized logistic regression (Firth, 1993) with *announcement condition* and *relevance* as independent factors. All continuous outcome variables were analyzed using two-way ANCOVA’s with delay announcement (4 types) and personal relevance (2 levels) as independent factors (Outcome ∼ Delay announcement + Personal relevance + Delay announcement × Personal relevance + Age + Gender). A bias corrected and accelerated bootstrap approach (1,000 bootstraps) was used as assumptions of ANOVA were not met. All analyses were corrected for age and gender. Due to small numbers, the categories *do not wish to disclose* and *other* were coded as missing from the gender variable before being included in the analyses. Analyses were performed using SPSS (v.29; IBM Corp., 2021) and R Statistical Software (v.4.5.1; R Core Team, 2025) using packages *logistf* (Heinze et al., 2025), *car* (Fox & Weisberg, 2019), *emmeans* (Lenth, 2025), *effectsize* (Ben-Shachar et al., 2020), and *boot* (Canty & Ripley, 2025).

### Research Transparency Statement

Data and analytical code are available upon reasonable request (Reference 61-2411). Use of the data for secondary analysis is subject to certain conditions (e.g., not to be used for commercial purposes).

## Results

### Sample

A total of 439 participants completed the survey of which 346 (79%) passed the manipulation check and included in the final sample. *M*_age_ was 55.94 years (*SD* = 16.67; unknown *n* = 9). In total, 160 participants indicated they were female (46%), 172 male (50%), five other (1%), eight did not want to disclose (2%), and one had a missing value. The sample distribution across conditions is presented in Table 2. The mean imagination (i.e., the extent to which participants could imagine themselves in the presented scenarios) across all eight scenarios was high (range 7.53–8.57; maximum = 10).

**Table 2:** Associations with suicide (%) per announcement type and personal relevance (*N* = 346)

Announcement type	Personal relevance	*n*	Association with suicide (%)
Collision person	Relevant	53	29 (55)
	Not relevant	42	19 (45)
	Total	95	48 (51)
Collision	Relevant	37	18 (49)
	Not relevant	42	6 (14)
	Total	79	24 (30)
Emergency services	Relevant	35	4 (11)
	Not relevant	40	8 (20)
	Total	75	12 (16)
Collision animal	Relevant	47	1 (2)
	Not relevant	50	0 (0)
	Total	97	1 (1)
Total sample	Relevant	172	52 (30)
	Not relevant	174	33 (19)
	Total	346	85 (25)

### Associations With Suicide

As shown in the logistic regression analysis results (Table 3), the odds of participants indicating suicide as the most probable cause of the delay was highest in the *collision with a person* condition. The *collision* condition was less likely to yield associations with suicide than the *collision with a person* condition (OR [95% CI] = 4.61 [1.7–13.89], *p* < .001), but more likely than in the *collision with animal* condition (OR [95% CI] = 0.05 [0.00–0.46], *p* < .001).

**Table 3:** Effect of announcement type and personal relevance on associations with suicide

Main and interaction effects	OR [95% CI]	*p*
Model 1: announcement type (ref. collision person)		
Collision	0.22 [0.07–0.59]	.002
Emergency services	0.32 [0.11–0.84]	.021
Collision animal	0.01 [0.00–0.09]	< .001
Model 2: personal relevance (ref. not personally relevant)		
Relevant	1.61 [0.70–375]	.26
Model 3: announcement type × personal relevance (ref. collision person × not personally relevant)		.036
Collision × relevant	2.88 [0.76–11.54]	.121
Emergency services × relevant	0.30 [0.06–1.31]	.109
Collision animal × relevant	2.06 [0.10–317.66]	.657
*Note*. Firth logistic regression. Models include gender and age as covariate. Ref. = reference group.		

There was a significant interaction effect between announcement and personal relevance on associations with suicide (Table 3). This was due to the difference in associations between the *emergency services* and *collision* condition in personally relevant and not relevant announcements (OR [95% CI] = 0.1, [0.02–0.51], *p* = .01), although wide confidence intervals around the odds ratios, especially for the *collision with an animal* condition, indicated imprecision.

### Emotional Impact of Announcements

Descriptive statistics of the continuous outcomes are presented in Table 4. With respect to emotional impact, a significant main effect of announcement condition was found for *anger toward the victim* and *empathy toward ProRail/Dutch Railways* (Table 5). In terms of personal relevance, significant main effects were found for all emotions except *empathy toward ProRail/Dutch Railways.* There was a significant interaction effect between announcement and relevance on *anger about the delay*. Post hoc pairwise comparisons showed that across all conditions, personally relevant announcements were associated with higher levels of anger than not personally relevant announcements (all *p*’s < .001). Within the not personally relevant condition, participants in the *emergency services* condition indicated significantly lower anger about the delay compared to the *collision with a person* condition (*p* = .018). There were no significant interaction effects for any of the other emotional impact variables.

**Table 4:** *M*s (SEs) for continuous outcome variables (range 1 [lowest impact/appreciation] – 10 [highest impact/appreciation])

Outcome variable	Personal relevance	Collision person *M* (*SE*)	Collision *M* (*SE*)	Emergency services *M* (*SE*)	Collision animal *M* (*SE*)
Anger about the delay	Relevant	4.12 (0.30)	4.51 (0.30)	4.71 (0.28)	4.17 (0.29)
	Not relevant	2.61 (0.40)	1.67 (0.28)	1.53 (0.29)	2.15 (0.35)
	Total	3.47 (0.25)	2.98 (0.26)	3.03 (0.27)	3.13 (0.25)
Sadness about the incident	Relevant	4.90 (0.37)	5.00 (0.47)	5.14 (0.34)	5.46 (0.33)
	Not relevant	4.80 (0.49)	4.11 (0.34)	4.05 (0.45)	3.95 (0.39)
	Total	4.86 (0.30)	4.53 (0.29)	4.57 (0.29)	4.67 (0.27)
Anger toward the victim	Relevant	3.02 (0.39)	2.70 (0.46)	3.46 (0.47)	1.78 (0.32)
	Not relevant	2.83 (0.46)	2.14 (0.38)	1.62 (0.32)	1.04 (0.27)
	Total	2.94 (0.30)	2.41 (0.30)	2.49 (0.30)	1.40 (0.21)
Acceptance of situation	Relevant	7.09 (0.30)	6.70 (0.33)	6.71 (0.28)	6.91 (0.30)
	Not relevant	7.64 (0.38)	7.52 (0.39)	7.87 (0.42)	8.16 (0.26)
	Total	7.33 (0.24)	7.14 (0.26)	7.32 (0.27)	7.56 (0.21)
Empathy toward ProRail/Dutch railways	Relevant	7.53 (0.31)	7.19 (0.38)	6.54 (0.41)	6.57 (0.25)
	Not relevant	7.45 (0.45)	6.76 (0.39)	6.38 (0.47)	5.67 (0.34)
	Total	7.49 (0.26)	6.96 (0.27)	6.46 (0.31)	6.11 (0.22)
Willingness to support other passengers	Relevant	4.81 (0.38)	5.70 (0.36)	5.20 (0.41)	5.85 (0.30)
	Not relevant	5.03 (0.50)	4.48 (0.42)	4.67 (0.50)	5.12 (0.37)
	Total	4.90 (0.30)	5.05 (0.29)	4.92 (0.32)	5.48 (0.24)
Announcement appreciation	Relevant	7.85 (0.27)	7.34 (0.28)	6.45 (0.31)	7.92 (0.29)
	Not relevant	7.78 (0.36)	7.98 (0.25)	8.04 (0.23)	8.22 (0.21)
	Total	7.82 (0.22)	7.69 (0.19)	7.30 (0.21)	8.07 (0.18)
*Note*. *SE* = standard error.					

**Table 5:** Main and interaction effects on continuous outcomes

Outcome variable	Delay announcement			Personal relevance			Delay announcement × Personal relevance		
	*F*(*df*)	*p*	η*p*^2^	*F*(*df*)	*p*	η*p*^2^	*F*(*df*)	*p*	η*p*^2^
Anger about the delay	0.44 (3)	.73	.004	105.71 (1)	**.001**	.254	2.99 (3)	**.031**	.028
Sadness about the incident	0.45 (3)	.72	.004	9.75 (1)	**.002**	.030	0.74 (3)	.528	.007
Anger toward the victim	5.10 (3)	**.002**	.047	9.36 (1)	**.002**	.029	1.32 (3)	.267	.013
Acceptance of situation	0.74 (3)	.53	.007	17.67 (1)	**<.001**	.054	0.40 (3)	.755	.004
Empathy toward ProRail/Dutch railways	6.15 (3)	**<.001**	.056	2.67 (1)	.103	.008	0.43 (3)	.731	.004
Willingness to support other passengers	0.86 (3)	.465	.008	6.19 (1)	**.013**	.020	1.07 (3)	.364	.010
Announcement appreciation	2.75 (3)	**.043**	.026	9.04 (1)	**.003**	.028	3.82 (3)	**.010**	.036
*Note*. Significant effects are bolded.									

Follow-up post hoc analyses of significant main effects showed that *anger toward the victim* was significantly lower in the *collision with an animal* condition than in any of the other three conditions (all Bonferroni-corrected *p* < .02). *Empathy toward Prorail/Dutch railways* was significantly higher in *the collision with a person* condition than in the *collision with an animal* and the *emergency services* condition (all Bonferroni-corrected *p* < .03). Regarding personal relevance, *anger toward the victim* and s*adness about the incident* were significantly higher when the imagined delay was personally relevant (*p* < .01). *Acceptance of the situation* was lower (*p* < .01) and *willingness to help fellow passengers* was higher when the delay was personally relevant (*p* = .01).

### Announcement Appreciation

There was a significant interaction effect between announcement condition and relevance on *announcement appreciation* (Table 5). Post hoc pairwise comparisons showed that within the personally relevant condition, participants exposed to the *emergency services* announcement indicated a significantly lower appreciation compared to *collision with a person* (*p* = .001) and *collision with an animal* (*p* = .002). Within the *emergency services* announcement, a significantly lower announcement appreciation was given in the personally relevant compared to the not personally relevant condition (*p* < .001).

## Discussion

The current study examined the degree to which different railway delay announcements bring about associations with suicide as well as emotional impact. The main findings were that the *collision* announcement was less likely to yield associations with suicide than the *collision with a person* announcement, but more likely to elicit suicide associations compared to the *emergency services* announcement. Compared to nonrelevant delays, personally relevant delays elicited more associations with suicide, stronger negative emotions, but also greater willingness to help fellow passengers.

In line with our first hypothesis (H1), we found that compared to the *collision with a person* announcement, associations with suicide were significantly lower in all other announcements. This result is in line with the UK-based study in which the message *collision with a person* was found to increase associations with suicide (compared to emergency services; Majava & McNaughton Nicholls, 2015). The authors argued that this message could potentially heighten public awareness of trains as a suicide method (Law et al., 2009) and shape perceptions of its commonness (Cialdini & Goldstein, 2004). The *collision* announcement was less likely to yield associations with suicide than the *collision with a person* announcement, but it was more strongly associated with suicide than the *emergency services* and *collision with an animal* announcements. It can be observed that there is a logical order in the size of the odds ratios by announcement that reflects how specific the announcement text is whereby the order of the odds ratios is inverse to the degree of specificity (0.01, 0.22, and 0.32; respectively, *collision with an animal → collision → emergency services*). This pattern underlines the validity of the measured associations.

In terms of emotional impact, the findings did not equivocally support H2 that the impact would be strongest in the *collision with a person* announcement. We did find that anger toward the victim was significantly higher in the *collision with a person* compared to the *collision with an animal* announcement but not compared to the *emergency services* or *collision* announcements. In addition, there was no significant difference between announcements on positive emotional impact.

The results showed that the personal relevance of the delay affected associations with suicide, confirming H3. In general, personally relevant delay announcements yielded more suicide associations than not personally relevant delay announcements. There was also support for H3, concerning positive and negative emotional impact. Personally relevant delay announcements were associated with more negative emotions than nonrelevant delay announcements. The current study adds nuance regarding the impact of personal relevance on anger about the delay. It seems that even if the delay announcement is not relevant, the *collision with a person* announcement evokes more anger than the *emergency services* condition. In addition, participants indicated more willingness to help fellow passengers when the announcement was personally relevant but were less accepting of the delay. Supporting H4, there was a higher level of announcement appreciation in the *collision with a person* condition compared to the *emergency services* condition, but only if the announcement was personally relevant.

Similar to the original study, our findings showed that the *collision with a person* condition had the highest odds of respondents indicating explicit associations with suicide. Previous findings showed that after *collision with a person*, participants were about nine times more likely to indicate suicide as the probable cause of the delay than the *emergency services* announcement (Van Leeuwen et al., 2020). These odds are higher than in the current study and higher when comparing to the *collision* condition. While we cannot make direct comparisons with the original study, we may speculate about the reasons for the subtle differences in likelihood of suicide associations. As the *collision with a person* announcement was replaced in 2021, it is possible that the public awareness and knowledge about the availability of trains as a means of suicide (Law et al., 2009) has reduced, thereby yielding less pronounced differences between announcement conditions. It is also possible that, as the previous announcement was in use for a longer period (> 10 years), it was more strongly embedded within public perception about the commonness of suicide by colliding with trains (Cialdini & Goldstein, 2004) compared to the current *collision* announcement that has been in use for just five years.

In the current study, personal relevance was found to influence associations with suicide whereby associations were more likely in personally relevant delays, except for the *emergency services* announcement. In addition, personal relevance impacted all emotions except *empathy toward ProRail/Dutch Railways*. This contrasts with the original study as personal relevance did not influence associations with suicide and the emotional impact effects were limited to *anger about the delay* and *anger toward the victim*. It is difficult to explain the differences between the two studies. In the context of the recent Dutch *On the Brakes! report* (Council for Health and Society [Raad voor Volksgezondheid en Samenleving], 2025), it could be speculated that societal expectations and demands have increased to the extent that delays to train journeys are perceived as a greater hindrance. Therefore, if a delay is personally relevant and interferes with a person’s plans, more negative emotions are experienced. At the same time, personally relevant delays yielded a greater willingness to help other passengers. The practical implications of this finding are limited as it is not possible for railway operators to adjust the delay announcement according to whether a delay is personally relevant to a passenger’s journey.

We replicated previous findings that the *emergency services* announcement was the least appreciated. It could be that this announcement is vaguer compared to the other announcements as there are many possible reasons for why and which emergency services are called out (for example the police, ambulance, fire engine). It might be worthwhile for railway companies to consider an adjustment to *emergency services,* for example by providing concrete information about how long the delay will last.

### Limitations

Although our study provides valuable insights, it also has limitations. First, the current experimental design limits the generalizability to real-life situations. Nevertheless, the fact that participants could generally imagine themselves well in the presented scenarios, as indicated by the high mean imagination ratings (>7.5 for all scenarios), means that a reasonable approximation was achieved. Second, the measure of associations with suicide assumes that the term *a person who was on the tracks on purpose* indeed reflects that participants thought the reason for the delay was a suicide. This assumption was necessary to test spontaneous associations with the different announcements. Third, to the best of our knowledge, the influence of suicide associations triggered by announcements leading to an act of suicide has not been studied. Nevertheless, some evidence suggests that media reports of suicide triggers copycat behavior (Niederkrotenthaler et al., 2009). Fourth, despite the use of the panel, our sample likely did not represent all individuals traveling by train (e.g., underrepresentation of students and individuals younger than 35 years). At the same time, this approach ensured a consistent study design and valid results, as it involved participants who travel regularly by train. Fifth, this study was conducted in the Dutch context, leading to the question of relevance to other countries. One might argue that in countries with lower population and railway density, people might be less likely to consider trains as a means of suicide. On the other hand, it is hard to find reasons why, on a psychological level, the difference between announcements would work differently in different contexts.

### Conclusion

Taking all findings together, we may conclude that *collision* is a preferable announcement because it triggers associations with suicide less than *collision with a person* condition. Alternatively, *emergency services* could also be preferred as this announcement did not differ significantly from *collision* on associations with suicide nor on emotional impact. However, the *emergency services* announcement is less appreciated. Overall, we recommend wording suicide-related delay announcements in a way that does not suggest suicide by train. From the perspective of suicide prevention on railways, we suggest that *collision* or *emergency services* are both appropriate announcements to use for suicide-related train delays.

## Notes

**Publication Ethics:** Informed consent was obtained from all participants included in the study. The Trimbos Institute’s Ethical Committee gave ethical approval for the study (Reference 202506) on April 23, 2025.

**Authorship:** Stephanie Leone, writing – original draft, data analyses; Felix Bolinski, writing, review & editing; Lonneke van Leeuwen, review & editing; Jeroen Bommelé, review & editing; Bart Hoogcarspel, conceptualization, review & editing; Anne C. Miers, conceptualization, writing, review & editing.

All authors approved the final version of the article.

**Open Science:** Data and analytical code are available upon reasonable request (Reference 61-2411). Use of the data for secondary analysis is subject to certain conditions (e.g., not to be used for commercial purposes).

The authors did not preregister this research in an independent, institutional registry. The research is a replication of an original study published in *Crisis* and the authors followed the analysis plan of the original study.
